# A database of submarine landslides offshore West and Southwest Iberia

**DOI:** 10.1038/s41597-021-00969-w

**Published:** 2021-07-16

**Authors:** Davide Gamboa, Rachid Omira, Pedro Terrinha

**Affiliations:** 1 Instituto Português do Mar e de Atmosfera – IPMA, I.P.; Rua C do Aeroporto, 1749-077 Lisbon, Portugal; 2 Instituto D. Luiz – IDL, Faculdade de Ciências da Universidade de Lisboa, Campo Grande, Edifício C8, Piso 3, 1749-016 Lisbon, Portugal

**Keywords:** Natural hazards, Physical oceanography

## Abstract

Submarine landslides are major geohazards occurring on distinct seabed domains ranging from shallow coastal areas to the deeper points of the ocean. The nature and relief of the seabed are key factors influencing the location and size of submarine landslides. Efforts have recently been made to compile databases of submarine landslide distribution and morphometry, a crucial task to assess submarine geohazards. The MAGICLAND (Marine Geo-hazards Induced by underwater Landslides in the SW Iberian Margin) database here presented contributed to that assessment offshore Portugal. Based on EMODnet bathymetric DEMs and GIS analysis, the morphometric properties of 1552 submarine landslides were analysed and wealth of 40 parameters was obtained. This dataset is now made available for the free use and benefit of the international marine community. Further contributions or analysis based on, and complementing the MAGICLAND database will be welcome.

## Abstract (summary)

**Table:** 

Measurement(s)	Morphometry of submarine landslides
Technology Type(s)	Geographic Information System
Sample Characteristic - Environment	continental margin • seamount
Sample Characteristic - Location	Northeast Atlantic Ocean coastal waters of the Iberian Peninsula

Machine-accessible metadata file describing the reported data: 10.6084/m9.figshare.14484342

## Background & Summary

Submarine mass movements are common occurrences on marine domains, from the shallow coasts to the deepest areas of the oceans^1^. The resulting landslides can be characterised by a variety of deposit features and morphologies, influenced by the mechanic properties of the original strata, the dynamics of the flow processes, regional geology and seismicity. Although singular massive deposits attract the attention for detailed studied, the regions where they occur can record geological evidence of hundreds or thousands of smaller-scale landslides, often poorly covered by available data and of limited focus of analysis.

Submarine landslides are a primary geohazard in marine environments. Tsunamis generated from landslides on the flank of subaerial topography flowing into the sea^2^, or from large collapses on fully submerged morphologic features^3^ are a major concern. Moreover, geotechnical installations and infrastructures resting on the seafloor such as submarine communication cables, pipelines or any purpose-build platform are sensible to mass movements^4^. Submarine landslides impact in marine biological communities, either by acting as habitat hotspots on their scars and remobilised elements or by disturbing and modifying seafloor ecology during emplacement^5^. Recognising submarine landslide extents has further political implication as these are used to set international ZEE boundaries under the definition of the UN Convention on Law of the Sea^6^. It is thus crucial to understand the distribution patterns and morphometric trends of submarine landslides according to the regional setting in which they occur, and aim to unravel insights on their causes and deposits^1,7^.

Efforts have been made to compile databases of submarine landslides to better understand their distribution and characteristics in marine settings around the world^1,7^. Regional compilations are available from the US Atlantic margin^8,9^, the Mediterranean Sea^10^, the Spanish margins^11^ or Australia^12^. Global data compilations have also allowed the comparison of landslides in distinct geological settings^7,13,14^. However, extensive submarine landslide characterisation is still lacking in many continental margins, and adequate characterisation depends on the quality of available data. Such is the case of the West and Southwest Iberian Margin, on the Northeast Atlantic Margin. This is an area of relevant geological risk, with frequent seismic activity resultant from the NW-ward collision of the African and European tectonic plates^15,16^. This has led to the occurrence of several high magnitude earthquakes (Mw > 7), of which the 1755 Lisbon Earthquake and tsunami is one of the major documented natural disasters^17^. Furthermore, the chains of large seamounts that occur in the area create major bathymetric features rising up to five kilometres above from the abyssal plain depths^16^, and are associated to intermediate to high seismicity, which is known to be a landslide trigger. Instability susceptibility studies conducted on the study area indicate that large extents of the continental slope and seamounts are prone to failure^18,19^. Yet, few submarine landslide studies exist, and these focused on specific case studies^3,20–22^.

**Fig. 1:**
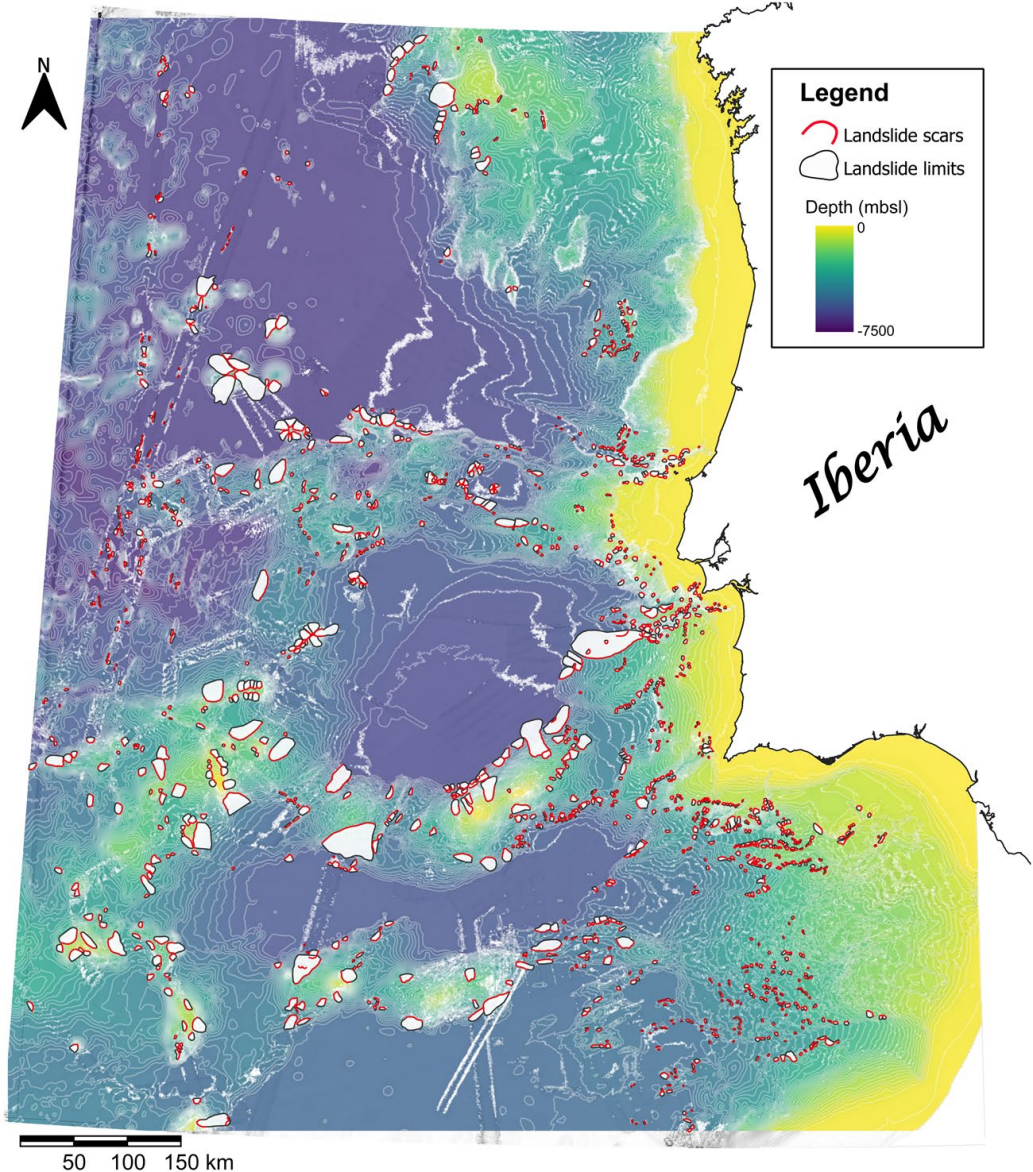
Map of the study area offshore West and Southwest Iberia shown as a blend of the bathymetry and slope gradient rasters. The red lines trace the limit of morphological scars identified on the EMODnet 2018 DEM. The grey polygons adjacent to the scars depict the landslide area, but only major ones are discernible at the scale presented. Contour lines were calculated from the DEM using a spacing of 100 m.

It is thus crucial and timely to provide a broader perspective of the distribution and morphometric trends of submarine landslides offshore Iberia. This work presents the MAGICLAND (Marine Geo-hazards Induced by underwater Landslides in the SW Iberian Margin) database, which covers the geographical area from 33° 45′ N to 43° N and from 6° 22′ W to 16° 15′ W, and compiles geomorphological data of 1552 submarine landslides based on the interpretation of DEM bathymetric grids provided by EMODnet^23^ (Fig. 1). Our results are crucial to understand the broad distribution of geohazards in the area, and aim to contribute to global efforts to compile landslide information in different geological and oceanic settings. This dataset is openly available through the Open Science Framework data repository^24^ for the use and benefit of the international marine and geohazard community. Further contributions or analyses based on, and complementing the MAGICLAND database will be welcome.

## Methods

**Fig. 2:**
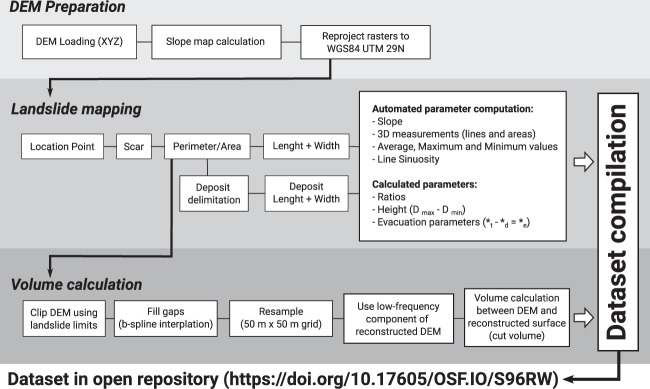
Workflow diagram of the submarine landslide mapping and analysis procedure used to compile the MAGICLAND database.

This section describes the methodology workflow used for data acquisition and preparation. This was set in three main stages, namely the Digital Elevation Model (DEM) data loading, the manual digitizing of landslide features, and volume calculation procedures. The main steps for each stage are summarised in Fig. 2.

### DEM loading and referencing

The mapping of scars and landslide features was based on the 2018 version of the EMODnet DTM (or DEM) for European seas covering the West and Southwest Iberian margins^23^ (Fig. 1), respectively referred to as WIM and SWIM. The EMODnet datasets result from the compilation of numerous bathymetric surveys made available by providers of 24 European countries, and include satellite derived bathymetry information derived from Landsat 8 imagery. Despite a general harmonization of the EMODnet data the density (and resolution) of its coverage is variable, a consequence of the data collection procedures and survey resources^19^. For this work, we used the XYZ data version of the EMODnet 2018 F3 DTM tile^23^ with a general 1/16 × 1/16 arc minutes grid, which at the latitude of Iberia corresponds to a resolution of approximately 115.6 m × 115.6 m. The XYZ data were loaded in the GIS software to produce DEM bathymetry and slope map rasters. Both rasters were reprojected from the WGS84 projection (EPSG:4326), used as default by EMODnet, to the WGS 84 UTM29N coordinate system (EPSG: 32629). All digitizing operations and morphometric measurements are based on the latter, with this also being the default projection system for the data provided in the repository^24^.

### Landslide morphometric mapping

**Fig. 3:**
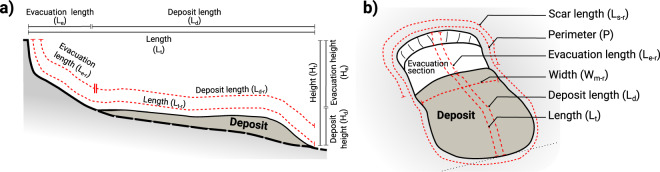
Schematic diagram of analysed landslide parameters. (**a**) Profile view along the landslide run-out, including the subdivision between the evacuation and deposit sections. Horizontal length lines at the top represent measurements based on a map perspective, while the dashed red lines represent realistic length measurements projected to the DEM 3D relief. The Height value represents the vertical different between the minimum depth at the scar upslope limit and maximum depth at the termination. (**b**) Perspective diagram representing the areal morphometric parameters analysed.

Mapping of the landslide morphological features observed on the DEMs was made using 2D and 3D visualisation perspectives on GIS software to delimit the scars and limits. Landslide morphometric mapping followed, as possible, established criteria^1^ for direct measurement features (Table 1 and Fig. 3), complemented by additional morphological and calculated parameters. Each landslides feature was identified with a unique reference ID (Scar_ID) to which all morphometric parameters recorded as point, line or polygon shapefiles were associated. Specifically, point features identify the location of each slide using an XYD reference; line features were used to trace the scar limit, and landslide length and width; and polygon features to delimit the landslide perimeter and the deposit section (when present or identifiable). The initial spatial association of these shapefiles used an automated proximity detection between the different features. The final merged shapefile was examined for consistency and for the correct match between the different elements, i.e., to make sure that all lines and polygons were associated to the same reference point and Scar_ID. This is a crucial quality check step as the automated process is prone to associate erroneous neighbour points in closely-spaced or overlapping features. The inaccurate records on line and polygon shapefiles derived from the automated merge were manually edited, and the corrected shapefiles were re-associated using the Scar_ID parameter. After the manual interpretation of the features on the DEM, automated processes were used to calculate additional parameters to populate the database. The parameter list and description are provided in Tables 1 and 2. In the instances where it was possible to identify the landslide deposit, this was delineated according to the morphological character displayed on the bathymetry and slope DEMs (Fig. 3). A second set of additional parameters was digitized for the deposit length, width, perimeter and area. The equivalent parameters were determined for the landslide evacuation section by subtracting the value of the deposit parameters from the total measurements.

**Table 1:** List of the morphometric parameters used in the MAGICLAND database.

Parameter	Unit	Description
Scar_ID	—	Unique identifier of the landslide feature
Confidence	—	Confidence of the landslide mapping quality - classified as 1, 2 or 3.
MultiScar	—	Y = mapped scar item includes coalesced scar; N = only a single scar is mapped
X	—	X position of the landslide data point in decimal degrees
Y	—	Y position of the landslide data point in decimal degrees
D	m	Reference value of depth below the seafloor of the landslide. All depth information records are presented as negative values.
L_t_	km	Total lenght mapped on the raster using a 2D map perspective
L_t-r_	km	“Realistic” total lenght derived from the projection of L_t_ on the 3D surface
L_t_/L_t-r_	%	Ratio, in percentage, between the L_t-r_ and L_t_ parameters
L_t-rAvSlp_	deg	Average slope grandient of L_t-r_ along the landslide remobilisation direction
W_m_	km	Width mapped on the raster using a 2D map perspective
W_m-r_	km	“Realistic” width value derived from the projection of W_m_ on the 3D surface
L_t_/W_m_	—	Length-Width ratio
L_t-r_/W_m-r_	—	Length-Width ratio calculated using the realistic measurement
P_t_	km	Perimeter of the full landslide-delimiting (evacuation and deposit, if present) polygon
A_t_	km^2^	Area of the landslide calculated within the polygon
A_t-r_	km^2^	Realistic surface area of the landslide derived from the 3D DEM
L_s_	km	Lenght of the landslide scar (alternatively, scar perimeter)
L_s-r_	km	Scar lenght measured along the 3D surface
Sin_s_	—	Sinuosity of the line delimiting the landslide scar
AvgD_s_	m	Avegerage depth of the landslide scar
D_min_	m	Minimum depth recorded, either at the lower limit of the scarp or toe of the deposit (if present)
D_max_	m	Maximum depth recorded, typically at the upslope limit of the scar.
H_t_	m	Height of the landslide, calculated as the difference between minimum depth at the downslope limit of the deposit or scar (D_min_), and maximum upslope limit of the scar (D_max_)
H_t_/L_t_	—	Height-Lenght ratio
L_t_/H_t_	—	Lenght-Height ratio
V	km^3^	Calculated volume evacuated by the landslide (equivalent to cut volume)
L_d_	km	Lenght of the deposit segment
L_d-r_	km	Realistic lenght of the deposit
L_d-rAvgSlp_	deg	Average slope of the deposit
AvgD_d_	m	Average depth of the deposit
H_d_	m	Height of the deposit
A_d_	km^2^	Area of the deposit
A_d-r_	km^2^	Realistic area of the deposit
P_d_	km	Deposit perimeter
L_e_	km	Lenght of the evacuation section
L_e-r_	km	Realistic lenght of the evacuation section
L_e-tAvgSlp_	deg	Average slope of the evacuation section
AvgD_e_	m	Average depth of the evacuation section
H_e_	m	Height of the evacuation section
A_e-r_	km^2^	Realistic area of the evacuation section

**Table 2:** Metadata summary table for specific data items and parameters.

Item/Parameter	Metadata
Bathymetry raster	Data downloaded from EMODnet data portal (https://www.emodnet-bathymetry.eu/data-products)
	Used the XYZ data version of the F3 panel, which was the base for a tight griding to create the work raster.
Slope raster	Calculated in QGIS using the “Slope” tool in the Raster terrain analysis options.
Reconstructed bathymetry raster	Derived from a copy of the bathymetry raster.
	Data clipped using the landslide-delimiting polygon (see file MagicLand-areas.shp) to delete all morphometric data within its limits.
	Surface reconstructed using a multilevel b-spline interpolation, resampled to a 50 m × 50 m grid.
	Pre-landslide morphology analysed using the low-frequency raster component resultant from the interpolation process.
XYD	References for each landslide reference point. X and Y indicate numeric coordinates based on the UTM 29 N grid. D is depth in metres. Location of the reference point is obtained as a centroid point derived from the landslide scar line.
Confidence	Interpreted-based attribute with value 1, 2 or 3.
	1- High confidence: landslide with clear scarps, limits and features (e.g., deposit blocks)
	2- Medium confidence: event boundaries partially smoothed or less clear, possibility of scar partially associated with turbiditic flows.
	3- Low confidence: poor limit definition due to smoothing, poor resolution or small size.
Length (total, deposit, evacuation segment, scar)	All length measurements are derived from bathymetric data, being as close to the maximum run-out path as possible.
	Digitization of the length object always followed a downslope direction, i.e., start on the upslope intersection with the scar limit and terminate at the deepest point identifiable. If bends are present on the flow path, the length was digitised as a polyline.
	Digitization was done on planview maps, and L_*_ was obtained from the horizontal line using the direct measurement tool from the GIS software.
	Realistic length measurements (L_*-r_) were obtained using an automated tool that projected the digitized line on the 3D DEM bathymetry surface. Sampling of values was taken along the full path of the line projected on the 3D surface.
	All length measurements of the evacuation segment were obtained by subtracting the deposit length from the total length.
Width	Width measurement followed, as possible, the recognition of the widest point of the landslide. In wide features following bent morphologies, the with was digitised as a polyline.
	Digitization was done on planview maps, and W_m_ was obtained from the horizontal line using the direct measurement tool from the GIS software.
	Realistic width measurements (W_m-r_) were obtained using an automated tool that projected the digitized line on the 3D DEM bathymetry surface. Sampling of values was taken along the full path of the line projected on the 3D surface.
Area (total, deposit, evacuation segment)	Total area measurements were made using a polygon to fully enclose all features associated with one landslide event. The deposit area was digitized where it was observable. The area of the evacuation segment was obtained by subtracting the deposit area from the total area.
	Realistic area measurements (A_*-r_) were obtained using an automated tool that calculated three-dimensional surface area of the area feature. This area is measured following all of the slopes of the terrain within the delimited polygon, as opposed to a planimetric area.
Volume	Volume was calculated using the QGIS Volume Calculation Tool plugin.
	The polygon input layer selected was MagicLand-areas.shp. Using this, the calculations were applied to every element mapped and the volume values automatically associated to it in the attribute table.
	The DEM height layer used was the reconstructed bathymetry raster (MagicLand-ReconstCover.tif)
	The base level DEM used was the bathymetry raster (MagicLand-BathymetryXYZ.tif).
	An Accurate Approximation Volume Calculation was selected, and the cut/fill counting method used. A sampling step of 50 was used for both X and Y.
	For the purposes of the database, the cut volume (equivalent to the volume removed from the evacuation area) was kept. The fill volume (roughly equivalent to the deposit) was discarded as calculating the deposit volume from two DEMs does not provide a minimally reliable value. Only through the use of subsurface data this can be achieved.

### Volume calculation

For volume calculation, a new DEM raster was calculated to represent the pre-landslide morphology (Fig. 2). To produce this reconstructed surface, a copy of the bathymetry raster was created. Next, the raster was clipped using the landslide limit polygon to remove all morphological data derived from each event. The data gaps were filled using a multilevel b-spline interpolation, further resampled to a 50 m × 50 m grid. The low-frequency raster component derived from this calculation was used as the model for pre-landslide morphology. All other interpolation product rasters were discarded as they do not produce accurate data for this objective.

The landslide evacuation volume calculations used the original bathymetry and the reconstructed morphology DEMs as base and top limiting surfaces, respectively. This operation used the QGis Volume Calculation Tool plugin, which allows the assignment of individualised polygons to delimit areas of operation. This feature greatly optimised the volume calculation task, especially taking into account the sample size. By constraining calculations within each individual landslide limit, the volume calculated for each feature was immediately added to the corresponding Scar_ID in the attribute table. For this work, we kept the values representative of the volume evacuated (cut) by the landslide. The landslide deposit (fill) volume calculated from the two used DEMs was discarded as it cannot be reliably estimated without subsurface data to identify the correct base of the deposit.

### Data preparation and visualisation

**Fig. 4:**
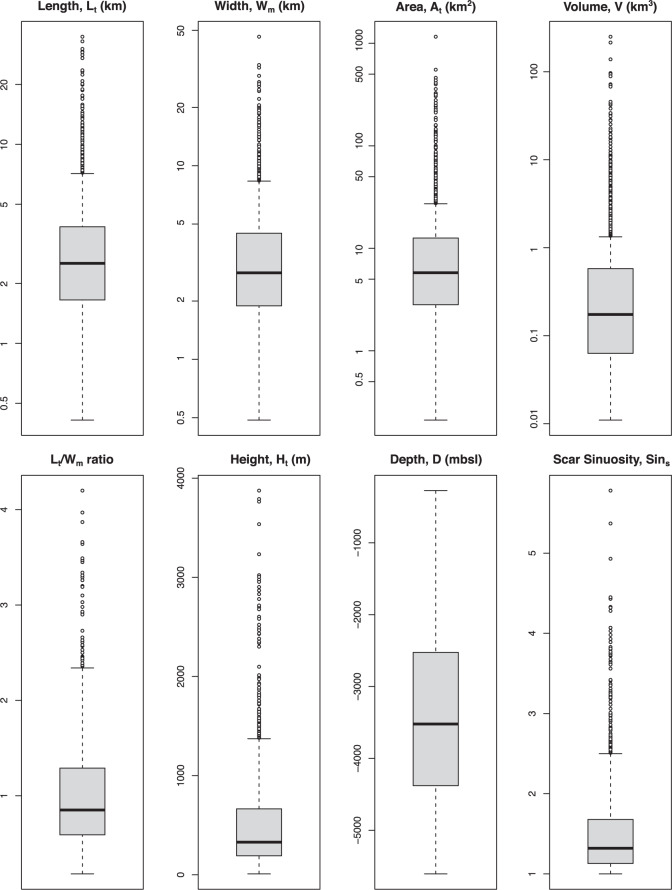
Boxplots summarizing the observations from eight selected landslide parameters. Logarithmic scales were used to represent the y-axis of length, width, area and volume for a clearer assessment of the distribution ranges.

The final data was compiled in a spreadsheet (MagicLand-Data.xlsx) using the Scar_ID as the data merging attribute. Sequential gaps in the numeric order of this attribute on the data provided are due to the manual removal of faulty entries which either had erroneous parameters or were outside of the target area. These erroneous data would ultimately skew any statistical analysis based on the dataset. Preliminary data for eight representative morphometric parameters are presented in Fig. 4. Logarithmic Y scales were used, and are recommended, for a better visualisation of parameters with very large ranges of values.

## Data Records

The MAGICLAND dataset^24^, available through the Open Science Framework (10.17605/OSF.IO/S96RW), includes a set of files with the shapefiles with the submarine landslide data (points, lines and polygons), relevant maps in GeoTIFF format, and data records in Microsoft Excel spreadsheets. The shapefiles relative to the landslide location points, scars and areas are included in the respective zip files. The GeoTIFF files correspond to the base bathymetry DEM (MagicLand-BathymetryXYZ.tiff), slope map (MagicLand-slopemap.tiff), reconstructed pre-landslide DEM (BathymReconstruct-Resample50x50_LowPassFilter.tiff) and the cover surface clipped to the landslide area (MagicLand-ReconstCover.tiff). Table 1 describing the morphometric parameters analysed and Table 2 with metadata descriptions are also included as in the dataset (MagicLand-Parameters.pdf and MagicLand-Metadata.pdf). The main data spreadsheet (MagicLand-Data.xlsx) includes the 1552 data entries and parameters. A subset corresponding to the morphometric properties of the deposit and evacuation regions is provided on a separate data file (MagicLand-DepositEvacData.xlsx).

## Technical Validation

The dataset presented exhibits sources of uncertainty inherent to distinct steps of the data compilation. These can be attributed to the base dataset used, to the manual interpretation of landslide scars, perimeter, length and width, and to the measurement accuracy.

### DEM resolution

The measurements derived from the DEM have an inherent data uncertainty derived from its resolution. Exact details for the spatial area covered in this analysis are not possible to be provided as the EMODnet data derives from the compilation of multiple surveys. This may increase inaccuracies and artifacts, a common issue with bathymetric data^25^. As the dataset used has a harmonised resolution of 115.6 m × 115.6 m, features smaller than the specified distances were not identifiable. The lower resolution areas of DEMs can also compromise the calculated slope values^26^ as no detailed morphologies are represented. On our dataset this issue has implications for the mapping and measurement of landslide parameters, particularly towards the western and southwestern limits. Here, the poorer data resolution is perceptible from the smoothed, less detailed contour lines (Fig. 1). Consequently, a lower number of landslides were mapped toward the western limits of the DEM.

### Interpreter bias and data limitations

The manual interpretation of the morphometric features consists of digitising lines and polygons close to the perceived morphological limits of the landslide on the DEM. This process is prone to variation between different individuals and can be influenced by factors such as map resolution and visualisation scale. While major parameters such as length or height tend to lead to low variability, others such as width or the delimitation of the evacuation and deposition areas are prone to higher interpreter variability^1^. This is prone to happen during replication of our work for parameters defined as single-value landslide features, such as width, that effectively change along its length. Nevertheless, the high number of samples likely attenuates the interpreter-derived variability and minimises deviations from the major statistical trends (Fig. 4). The delimitation of the landslide deposit, when identifiable, is likely underestimated as the DEM only expresses seafloor morphologies. Consequently, when the landslide deposit is partially or fully buried, the deposit and full landslide real length may be higher than the values recorded.

### Volume calculation

The accurate volume calculation of the 1552 landslides presented the biggest challenge as we tried to use a uniform method that is applicable to all landslides at once. Interpolated top surfaces across the landslide scar area is a method successfully used to reconstruct top pre-landslide morphologies in previous studies^9^, and this was suitable to use in our database objectives. While on longitudinal sections the reconstructed surface has adequate matches with the landslide limits on the bathymetry DEM, transverse sections clipped to the landslide limits may intersect the sidewall at points below its apex. Thus, absolute volume calculation can be underestimated. The reconstruction may also present limitations for smaller landslides in low slope gradient areas. However, this compromise is required to allow the swift volume computation for all elements identified. Furthermore, it should not significantly affect comparative analysis of relative landslide volume magnitude between distinct examples or locations.

### Measurement usage in 3D

The standard procedure to digitise lines and polygons on GIS is based on 2D map projections, with subsequent spatial measurements being primarily derived from planimetric perspectives. This procedure was the base to obtain the landslide total length (L_t_), width (W_m_) or area (A_t_) parameters. However, the effect of slope gradient on distance and area calculations is relevant and it is crucial to estimate it to obtain, for instance, the realistic submarine landslide runout length. To mitigate this issue, the lines and polygons of landslide features mapped using the planimetric perspective were projected and recalculated over the 3D DEM. Thus, an additional set of length, width and area parameters was obtained for the full landslide and for its deposit and evacuation subsections. The parameters derived from the projection on the 3D surface are identified as *_*-r_ (see Table 1 for the full listing) where the r stands for “realistic”, or at least close to it given the inherent data resolution and uncertainties.

**Fig. 5:**
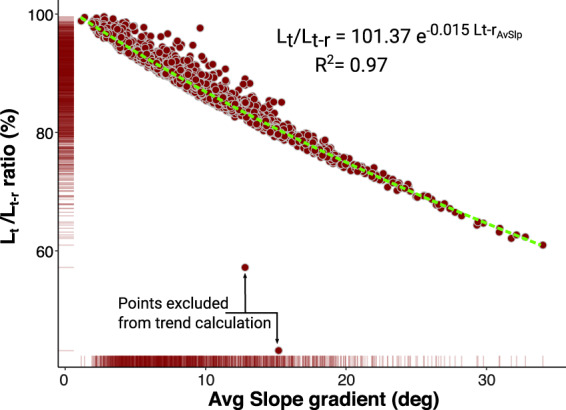
Scatter plot representing the impact of slope gradient on the L_t_ and L_t-r_ measurement. The L_t_/L_t-r_ ratio indicates how much the planimetric and 3D-projected length measurements differ, with values closer to 100% indicating a minimum or no difference. As shown, the lower the slope gradient, the lower the L_t_ diverges from the real topographic value L_t-r_. The fringes adjacent to the plot axis represent the frequency of registered values.

Figure 5 illustrates the aforementioned effect of slope gradient on the length measurement of morphological features using the L_t_/L_t-r_ ratio and the average gradient along the landslide runout vector. Very low slope angles will have minimal impact on the length measurements, but for angles of 15 degrees L_t_ can be 20% shorter than the L_t-r_ measurement. Towards the steeper slope values of our sample, of circa 30 degrees, this effect can lead to L_t_ measurements 35% shorter than the L_t-r_. The same principle is valid for width, perimeter and area measurements.

## Usage Notes

All researchers interested in submarine geomorphology, landslides and GIS are free to use the datasets provided at will, with appropriate acknowledgement of the source. The data provided in the repository allows an immediate reproducibility of the results and opens possibilities for further statistical analysis and integration with other databases – being that for individual research items or integration at wider scale. The majority of the work was produced using QGIS v3.14, but all items are importable to any GIS software of choice. Despite the high number of features mapped, there are many more occurrences of landslide and mass-movement features passive of being mapped. Further versions of the MAGICLAND database will make efforts to integrate subsurface information and higher detail metrics when higher resolution bathymetric data is available. Researchers are welcome to contribute to the development of this dataset as deemed fit, either by improving knowledge of the mapped features or adding new ones.

## Data Availability

No customized code was produced to prepare or analyse the dataset.
